# Age-dependent association of body mass index and obstructive sleep apnea severity in women

**DOI:** 10.1007/s11325-026-03849-y

**Published:** 2026-10-31

**Authors:** Unjung Cho, Chang-Hoon Lee, Mark Dyken, Rachel Immen, KyoungBin Im

**Affiliations:** 1 UCI Health Sleep Disorders Center, University of California, 20350 SW Birch Street, Suite 100, CA 92660 Newport Beach, United States of America; 2 Department of Psychiatry and Human Behavior, University of California, Irvine, CA United States of America; 3 Division of Pulmonary and Critical Care Medicine, Department of Internal Medicine, Seoul National University Hospital, Seoul, Republic of Korea; 4 The Department of Neurology, University of Iowa Hospitals and Clinics, Iowa City, IA United States of America; 5 Carle BroMenn Medical Center, Bloomington, IL United States of America; 6 Department of Psychiatry, Inha University Hospital, Inha University College of Medicine, Incheon, Republic of Korea

**Keywords:** Obstructive sleep apnea, Body mass index, Aging, Sex differences, Women

## Abstract

**Purpose:**

Obstructive sleep apnea (OSA) is influenced by body mass index (BMI), age, and sex. Although obesity is a major risk factor for OSA, the strength of the association between BMI and OSA severity may vary across demographic groups. We investigated whether age modifies the relationship between BMI and OSA severity in men and women.

**Methods:**

We performed a cross-sectional analysis of adults who underwent polysomnography at a university-based sleep laboratory. The relationship between BMI and apnea-hypopnea index (AHI), including supine AHI, was evaluated. Participants were categorized into younger and older age groups (< 55 vs. ≥55 years). Linear regression models with interaction terms were used to assess whether age modified the association between BMI and OSA severity.

**Results:**

A total of 752 participants were included. The associations of BMI categories with normalized AHI and normalized supine AHI were weaker in older women than in younger women (*P* for interaction = 0.03 and 0.02, respectively), whereas no significant interaction by age group was observed in men. Results were similar for log-transformed BMI (*P* for interaction = 0.03 for both in women). However, the three-way interaction among BMI, age group, and sex was not significant.

**Conclusions:**

The association between BMI and OSA severity was attenuated in women aged 55 years or older compared with younger women. Because a sex difference in this age-related attenuation was not statistically confirmed, these findings should be interpreted with caution. They suggest that BMI alone may be a less informative indicator of OSA severity in older women.

**Supplementary Information:**

The online version contains supplementary material available at https://doi.org/10.1007/s11325-026-03849-y.

## Introduction

Obstructive sleep apnea (OSA) is a prevalent sleep disorder characterized by recurrent upper airway obstruction during sleep, resulting in intermittent hypoxemia and sleep fragmentation. The prevalence and severity of OSA are influenced by multiple factors, including obesity, age and sex [1].

Obesity is one of the strongest and most modifiable risk factors for OSA, with increasing body mass index (BMI) associated with greater OSA risk and severity [2, 3]. Aging is another important contributor to OSA. The prevalence of OSA increases with age, likely because of age-related changes in upper airway anatomy, neuromuscular control, and ventilatory regulation [4]. In addition, substantial sex differences in OSA prevalence have been consistently observed, with men exhibiting a two- to threefold higher risk than women [5, 6].

Interestingly, the sex difference in OSA prevalence becomes less pronounced with advancing age, largely because OSA prevalence increases among older women [7]. Several studies have suggested that menopause and associated hormonal changes may contribute to this increase, independent of obesity [8, 9]. Declining estrogen and progesterone levels have been hypothesized to reduce upper airway muscle tone and alter ventilatory control, thereby increasing susceptibility to OSA [10, 11].

Previous studies have reported that the relationship between obesity and OSA may differ in older adults, with some showing a weaker association between excess body weight and OSA than is observed in younger populations [12]. These findings suggest a potential interaction between age and BMI in determining OSA risk and severity. However, whether this interaction differs between men and women remains unclear. Most prior studies have focused primarily on menopause-related mechanisms in women [9, 13], making it difficult to distinguish the effects of aging from sex-specific biological factors.

Aging may also influence positional aspects of OSA. Age-related reductions in upper airway muscle tone can increase airway collapsibility and enhance the effects of gravity on upper airway patency during sleep [4, 14]. Recent studies suggest that older adults are more likely to exhibit positional OSA, with apnea-hypopnea index (AHI) increasing disproportionately in the supine position compared with non-supine positions [15, 16]. Given that BMI is a major modifiable determinant of OSA, it is important to determine whether the relationship between BMI and OSA severity differs according to age, sex, and sleep position.

Therefore, this study investigated whether age modifies the association between BMI and OSA severity in both men and women. We specifically examined whether the relationship between BMI and OSA severity, including supine OSA severity, differs between younger and older adults and whether these age-related differences vary by sex.

## Materials and methods

### Participants

This cross-sectional study was conducted at the Sleep Disorders Center at the University of Iowa Hospitals and Clinics (UIHC), analyzing data from adults who underwent polysomnography (PSG) between July 2011 and June 2012. Those who had a total sleep time of less than 30 min and those with a total sleep time in the supine position of less than 15 min were excluded. A total of 752 participants were included in the final analysis. The study was approved by the Institutional Review Board (IRB) at The University of Iowa (IRB number 201210723). A Data Transfer and Use Agreement was mutually approved by both IRBs of The University of Iowa and the University of California Irvine (J543200-DUA).

### Measurements

Demographic information, including age, sex, weight, height, body mass index, and neck circumference, along with medical comorbidities such as diabetes mellitus, hypertension, coronary artery disease, congestive heart failure, chronic obstructive pulmonary disease, depression, and stroke and/or transient ischemic attack, was collected at the time of PSG. The Epworth Sleepiness Scale (ESS) was also assessed on the same day.

Participants were categorized into younger (< 55 years) and older (≥ 55 years) groups. The age threshold of 55 years was selected because it approximates the upper end of the menopausal transition for most women and has been used in epidemiologic studies examining age-related changes in health outcomes [17]. Sensitivity analyses were also performed using an alternative age cutoff of 50 years [18]. BMI was analyzed both as a continuous variable (log-transformed) and categorical groups: <25 kg/m², 25–29.9 kg/m², 30–39.9 kg/m², and ≥ 40 kg/m².

### Polysomnography results and outcomes

PSG data were retrospectively obtained from the Sleep Disorders Center at the UIHC. Overnight PSG recordings included continuous monitoring of electroencephalogram, electrooculogram, electromyogram, electrocardiogram, airflow (measured using a pressure transducer and thermistor), chest and abdominal wall movements, along with synchronized audio-video recordings. Certified technologists and physicians, accredited by the American Academy of Sleep Medicine (AASM) scored the PSG data according to the AASM guidelines [19] in place at the time of the study, assessing sleep stages, respiratory events, and movement disorders. AHI was calculated as the total number of apneas (defined as complete cessation of airflow for ≥ 10 s) and hypopneas (characterized by reduced respiratory effort accompanied by ≥ 4% oxygen desaturation) per hour of sleep. The primary outcome was AHI, including total and supine AHI (AHI during supine position), as a measure of OSA severity [20, 21].

### Statistical analysis

Continuous variables were compared between younger and older participants within each sex using Student’s t-tests. The assumption of equal variances was assessed using Levene’s test. Because AHI and supine AHI were right-skewed, they were also summarized as median (interquartile range) and compared using the Wilcoxon rank-sum test and the Kruskal–Wallis test. Categorical variables were compared using the chi-square test or Fisher’s exact test when expected cell counts were low. One-way analysis of variance (ANOVA) was used to assess differences among the four groups (younger men, older men, younger women, and older women), followed by Tukey’s honestly significant difference (HSD) test for pairwise comparisons.

BMI exhibited a right-skewed distribution and was therefore log-transformed for analyses treating BMI as a continuous variable. AHI measures, including total AHI and supine AHI, were also right skewed and contained a substantial proportion of zero values. Because log transformation, Box-Cox transformation, and Yeo-Johnson transformation did not adequately normalize these distributions, rank-based inverse normal transformation (INT) was applied to generate normalized AHI and normalized supine AHI variables (Supplementary Table 1) [22, 23].

To evaluate whether age modified the association between BMI and OSA severity, linear regression models incorporating interaction terms were used. Separate models were constructed using normalized AHI and normalized supine AHI as dependent variables. BMI was analyzed both as a categorical variable (< 25, 25–29.9, 30–39.9, and ≥ 40 kg/m²) and as a continuous log-transformed variable. Interaction terms between age group (< 55 vs. ≥55 years) and BMI were included to evaluate effect modification, and corresponding interaction coefficients (β) with 95% confidence intervals (CIs) and *P*-values were reported. In the interaction models, BMI category was applied as an ordinal score (1–4) to test for a linear trend across categories. Because the dependent variables were rank-based inverse normal scores, regression coefficients are expressed in standard deviation units of the normalized outcome. To examine whether the effect modification by age differed between men and women, a BMI × age group × sex three-way interaction term was additionally tested in a pooled model including both sexes. A two-sided *P* value < 0.05 was considered statistically significant. All statistical analyses were performed using Stata 17.0 (StataCorp, College Station, TX, USA).

## Results

### Demographic and clinical characteristics

**Fig. 1:**
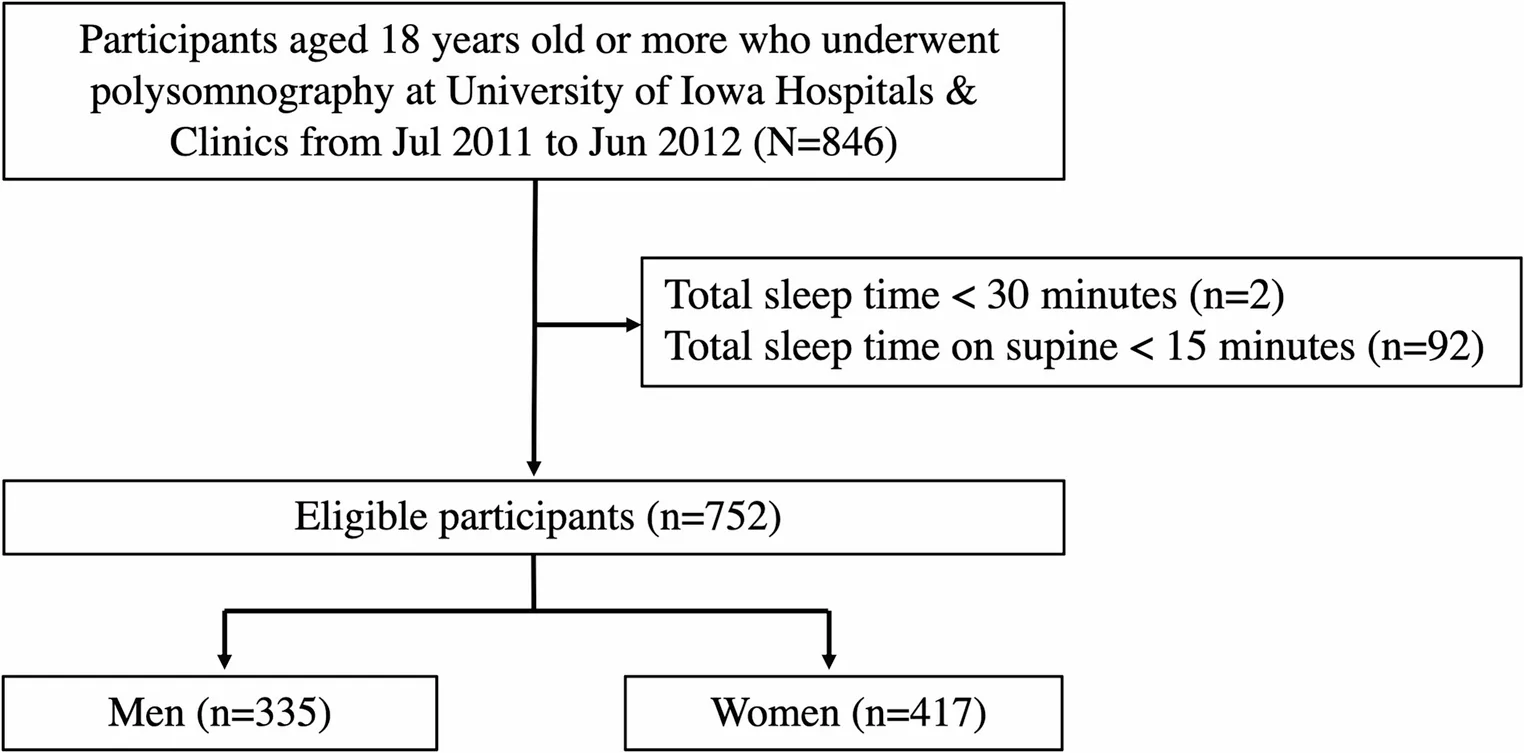
Flowchart of including participants

Of the total 846 participants enrolled, those with a total sleep time of less than 30 min (*n* = 2) and those with a total sleep time in the supine position of less than 15 min (*n* = 92) were excluded. A total of 335 men and 417 women were included in the final analysis (Fig. 1). The demographic and clinical characteristics of the study population are summarized in Table 1. Participants were stratified by age into younger (< 55 years) and older (≥ 55 years) groups, as well as by sex. No significant differences were observed in body mass index (BMI) between younger and older men (32.6 ± 7.8 vs. 31.0 ± 7.1, *P* = 0.06) or women (35.1 ± 10.4 vs. 33.5 ± 8.5, *P* = 0.11). Similarly, neck circumference did not differ significantly between age groups in either sex (*P* > 0.05). The prevalence of hypertension was also significantly higher in the older group for both men (37.2% vs. 69.8%, *P* < 0.001) and women (44.0% vs. 64.1%, *P* < 0.001). Additionally, Epworth Sleepiness Scale (ESS) scores were significantly lower in the older women than in the younger women (10.9 ± 5.2 vs. 9.4 ± 5.4, *P* = 0.01), suggesting a potential reduction in subjective daytime sleepiness with age.

**Table 1:** Comparison of demographic and comorbidities between groups

	Men			Women		
	Age < 55 YO	Age ≥ 55 YO	*P*-value	Age < 55 YO	Age ≥ 55 YO	*P*-value
N	219	116		300	117	
Age, years (SD)	40.1 (9.7)	63.4 (7.0)	< 0.001 ^*^	40.7 (9.6)	62.2 (6.5)	< 0.001^*^
Body Mass Index, kg/m²	32.6 (7.8)	31.0 (7.1)	0.06	35.1 (10.4)	33.5 (8.5)	0.11
Epworth Sleepiness Scale	9.9 (4.9)	9.1 (4.8)	0.18	10.9 (5.2)	9.4 (5.4)	0.01^*^
Neck Circumference, cm	42.9 (4.3)	42.6 (4.2)	0.56	38.2 (4.3)	37.8 (3.7)	0.38
Hypertension, N (%)	81 (37.2)	81 (69.8)	< 0.001^*^	132 (44.0)	75 (64.1)	< 0.001^*^
Diabetes	31 (14.2)	32 (27.6)	0.004 ^*^	49 (16.3)	23 (19.7)	0.51
Coronary artery disease	10 (4.6)	21 (18.1)	< 0.001^*^	9 (3.0)	6 (5.1)	0.45
Congestive heart failure	8 (3.7)	7 (6.0)	0.41	13 (4.3)	4 (3.4)	0.79
Chronic obstructive pulmonary disease	44 (20.1)	25 (21.6)	0.86	73 (24.3)	25 (21.4)	0.61
Stroke/ Transient ischemic attack	3 (1.4)	2 (1.7)	1.00	5 (1.7)	4 (3.4)	0.28
Depression	62 (28.3)	26 (22.4)	0.30	166 (55.3)	61 (52.1)	0.63

Data are presented as mean (SD) or No. (%). * =*P* < 0.05. *YO* Years Old

### Polysomnographic findings

Polysomnographic parameters are presented in Table 2. Total sleep time (TST) was significantly shorter in the older group for both men (341.5 ± 80.9 vs. 312.0 ± 78.3 min, *P* = 0.002) and women (360.5 ± 87.1 vs. 340.0 ± 66.8 min, *P* = 0.02). Sleep efficiency was also significantly lower in the older men (73.8 ± 17.1% vs. 80.5 ± 14.2%, *P* < 0.001) and the older women (78.3 ± 12.7% vs. 81.8 ± 12.3%, *P* = 0.01), indicating decreased sleep efficiency with age. AHI was significantly higher in the older women than in the younger women (mean 8.8 ± 7.4 vs. 5.5 ± 8.1 events/h; median 6.3 [IQR 2.7–14.1] vs. 2.6 [0.3–6.5] events/h, *P* < 0.001). However, the mean difference was not statistically significant in men (12.8 ± 15.3 vs. 10.0 ± 12.7 events/h, *P* = 0.08). Zero values were observed in 10.1% of participants for total AHI and 13.4% for supine AHI, most frequently in younger women (Table 2). All participants with zero supine AHI had at least 15 min of supine sleep (median 141 min [IQR 60–230]).

**Table 2:** Comparison of polysomnographic sleep parameters between groups

	Men			Women			One-way ANOVA *p*-value
	Age < 55 YO	Age ≥ 55 YO	*p*-value	Age < 55 YO	Age ≥ 55 YO	*p*-value	
N	219	116		300	117		
Total sleep time, min (SD)	341.5 (80.9)	312.0 (78.3)	0.002^*^	360.5 (87.1)	340 (66.8)	0.02^*^	< 0.001^*^
Supine Sleep Time, min (%)	181.8 (53.1)	161.7 (52.2)	0.11	185.9 (51.7)	170.7 (49.4)	0.20	0.17
Sleep Efficiency, % (SD)	80.5 (14.2)	73.8 (17.1)	< 0.001^*^	81.8 (12.3)	78.3 (12.7)	0.01^*^	< 0.001^*^
AHI, events/h, mean (SD)	10.0 (12.7)	12.8 (15.3)	0.08	5.5 (8.1)	8.8 (7.4)	< 0.001^*^	< 0.001^*^
Median (IQR)	5.2 (1.6–14.9)	8.3 (3.6–17.2)	0.005^*†^	2.6 (0.3–6.5)	6.3 (2.7–14.1)	< 0.001^*†^	< 0.001^*‡^
AHI = 0, No. (%)	12 (5.5)	6 (5.2)	1.00	54 (18.0)	4 (3.4)	< 0.001^*^	
Supine AHI, mean (SD)	18.2 (23.7)	24.1 (25.7)	0.04^*^	8.7 (12.9)	15.8 (17.2)	< 0.001^*^	< 0.001^*^
Median (IQR)	8.9 (2.2–24.3)	13.3 (4.4–40.5)	0.013^*†^	3.8 (0.4–11.6)	9.6 (3.5–24.8)	< 0.001^*†^	< 0.001^*‡^
Supine AHI = 0, No. (%)	16 (7.3)	9 (7.8)	1.00	66 (22.0)	10 (8.5)	0.001^*^	
REM supine AHI	16.7 (24.9)	15.7 (21.0)	0.72	13.6 (21.0)	23.0 (27.4)	< 0.001^*^	0.003^*^
NREM supine AHI	17.2 (24.1)	23.2 (26.6)	0.04^*^	7.1 (12.8)	13.6 (17.3)	< 0.001^*^	< 0.001^*^
Non supine AHI	4.8 (11.7)	4.4 (6.8)	0.69	3.2 (7.8)	3.8 (5.0)	0.44	0.17
REM non-supine AHI	6.0 (14.6)	6.8 (12.2)	0.65	5.5 (13.6)	8.1 (17.2)	0.10	0.39
NREM non-supine AHI	4.3 (12.0)	3.5 (6.5)	0.49	2.4 (7.3)	2.9 (4.3)	0.47	0.07
PLM index	9.1 (17.2)	20.3 (30.8)	< 0.001^*^	8.6 (20.0)	15.9 (25.5)	0.002^*^	< 0.001^*^

Data are presented as mean (SD), median (IQR), or No. (%). * = *P* < 0.05. † Wilcoxon rank-sum test; ‡ Kruskal–Wallis test; proportions of zero values were compared using Fisher’s exact test. *AHI *Apnea-Hypopnea Index, *IQR* Interquartile Range, *REM *Rapid Eye Movement, *NREM* Non-Rapid Eye Movement, *PLM* Periodic Limb Movement, *YO* Years Old

### Effect of age on BMI-OSA

**Fig. 2:**
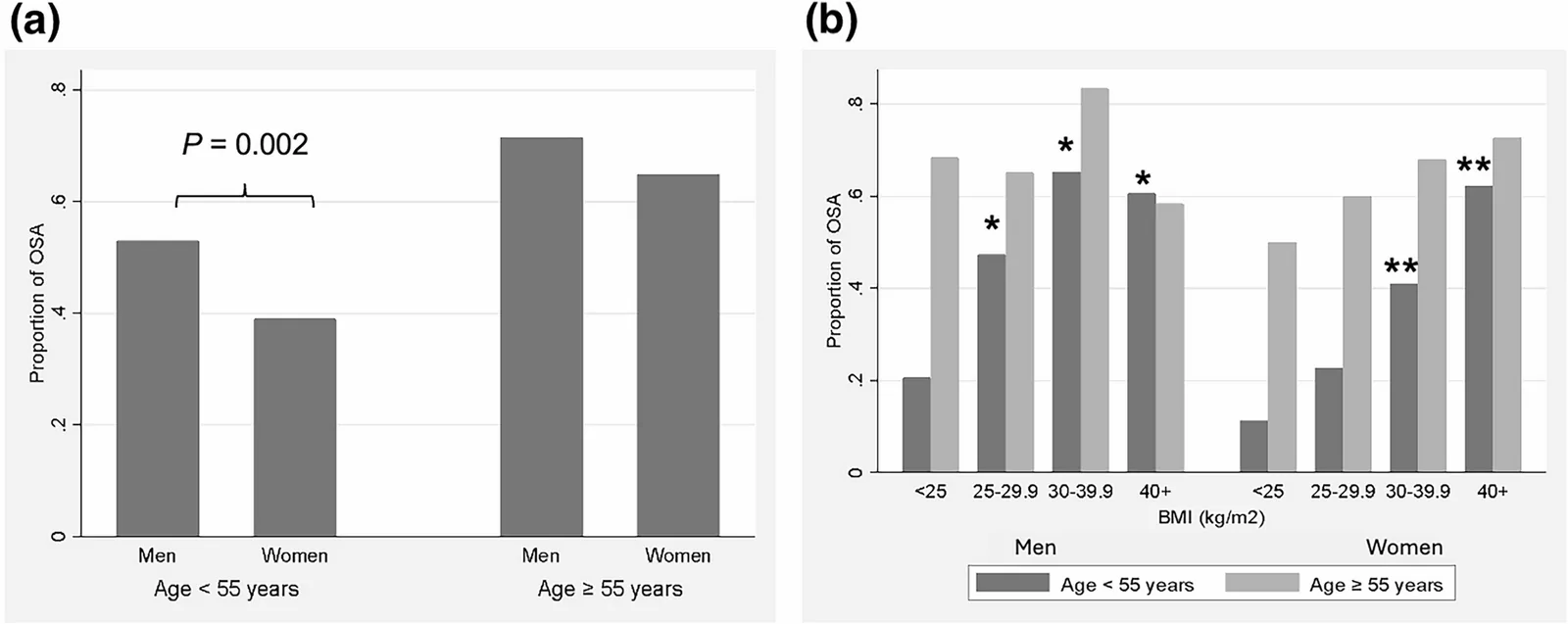
Proportion of OSA according to sex, age, and BMI. (**a**) Proportion of OSA according to sex and age group. In the younger age group, the proportion of OSA was higher in men than in women (53.0% vs. 39.0%, *P* = 0.002), whereas no significant difference was observed between older men and women (71.6% vs. 65.0%). (**b**) Proportion of OSA across BMI categories according to sex and age group. Within the younger age group of each sex, the proportion of OSA was higher in groups with higher BMI compared with the < 25 kg/m² group. In the older age group, the proportion of OSA did not differ significantly across BMI categories. * *P* < 0.05 compared with BMI < 25 kg/m² in men aged < 55 years. ** *P* < 0.05 compared with BMI < 25 kg/m² in women aged < 55 years

Younger men had a higher proportion of OSA than younger women (53.0% vs. 39.0%), while there was no significant difference between older men and older women (71.6% vs. 65.0%). Within the younger age group of each sex, the proportion of OSA was higher in groups with higher BMI compared to the < 25 kg/m2 group. Younger men had an OSA proportion of 20.6% in the BMI < 25 kg/m² group, 47.4% in the 25–29.9 kg/m² group, 65.3% in the 30–39.9 kg/m² group, and 60.6% in the ≥ 40 kg/m² group. A similar trend was observed in younger women, with OSA proportions of 11.4% (BMI < 25 kg/m²), 22.8% (BMI 25–29.9 kg/m²), 41.0% (BMI 30–39.9 kg/m²), and 62.2% (BMI ≥ 40 kg/m²). However, in the older age group, there was no significant difference in OSA proportion across BMI groups (men: 68.4% in BMI < 25 kg/m2, 65.1% in BMI 25–29.9 kg/m2, 83.3% in BMI 30–39.9 kg/m2, and 58.3% in BMI 40 + kg/m2; women: 50.0% in BMI < 25 kg/m2, 60.0% in BMI 25–29.9 kg/m2, 67.9% in BMI 30–39.9 kg/m2, and 72.7% in BMI 40 + kg/m2) (Fig. 2). These results remained consistent when older age was defined as ≥ 50 years (Supplementary Fig. 1).

**Fig. 3:**
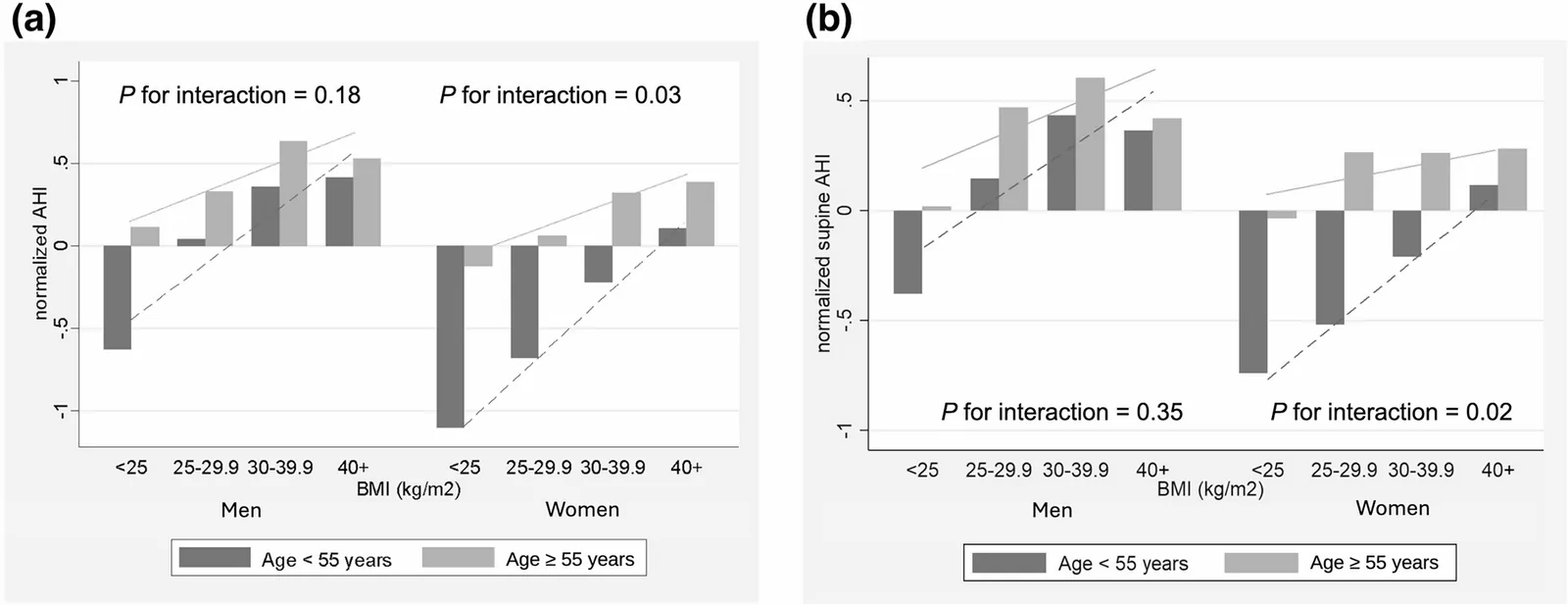
Normalized AHI and supine AHI according to BMI group (**a**) The association between normalized AHI and BMI groups according to sex and age. (**b**) The association between normalized supine AHI and BMI groups according to sex and age. Normalized AHI and normalized supine AHI increased with higher BMI categories in both men and women. In women, a significant interaction between age group and BMI category was observed, indicating that the association between BMI and AHI varied by age. In men, no significant interaction was found

Normalized AHI and normalized supine AHI showed positive associations with higher BMI categories in both men and women. The BMI–AHI slopes within each age and sex subgroup and the differences between the slopes of the older and younger groups are summarized in Table 3. The BMI × age group interaction coefficient in each sex-specific model corresponds to this difference. A significant interaction between age group and BMI category was observed for normalized AHI and supine AHI in women (β for interaction = − 0.22, 95% CI − 0.42 to − 0.03, *P* = 0.03; and β = −0.22, 95% CI − 0.40 to − 0.03, *P* = 0.02, respectively), but not in men (β = −0.16, 95% CI − 0.40 to 0.08, *P* = 0.18; and β = −0.10, 95% CI − 0.33 to 0.12, *P* = 0.35) (Fig. 3; Table 3). However, the BMI category × age group × sex three-way interaction was not statistically significant for either normalized AHI (β = −0.06, 95% CI − 0.36 to 0.24, *P* = 0.69) or normalized supine AHI (β = −0.11, 95% CI − 0.40 to 0.17, *P* = 0.43), indicating that the difference in effect modification by age between men and women was not statistically significant. In sensitivity analyses in which the older age group was defined as 50 years or older, the results for normalized supine AHI were similar (women: β = −0.34, 95% CI − 0.49 to − 0.18, *P* < 0.001; men: β = −0.17, 95% CI − 0.38 to 0.03, *P* = 0.09), except that the interaction for normalized AHI was also significant in men (β = −0.27, 95% CI − 0.49 to − 0.05, *P* = 0.02; women: β = −0.30, 95% CI − 0.47 to − 0.14, *P* < 0.001). The three-way interaction with sex remained non-significant (*P* = 0.82 for normalized AHI and *P* = 0.20 for normalized supine AHI) (Supplementary Fig. 2).

**Table 3:** BMI–AHI slopes by age and sex subgroup and absolute differences between slopes

Outcome (per 1-category higher BMI)	Sex	Slope, < 55 y (95% CI)	Slope, ≥ 55 y (95% CI)	Absolute Difference (95% CI)	*P*
Normalized AHI	Men	0.35 (0.22, 0.49)	0.19 (− 0.00, 0.39)	−0.16 (− 0.40, 0.08)	0.18
	Women	0.41 (0.31, 0.50)	0.18 (0.01, 0.35)	−0.22 (− 0.42, − 0.03)	0.03^*^
Normalized supine AHI	Men	0.27 (0.14, 0.39)	0.16 (− 0.02, 0.34)	−0.10 (− 0.33, 0.12)	0.35
	Women	0.29 (0.20, 0.38)	0.07 (− 0.09, 0.23)	−0.22 (− 0.40, − 0.03)	0.02^*^

Slopes are expressed in SD units of the rank-based inverse normal transformed outcome. Absolute Difference = BMI × age group interaction coefficient from sex-specific linear regression models. * = *P* < 0.05. *BMI *Body Mass Index, *AHI* Apnea-Hypopnea Index, *CI* Confidence Interval, *SD* Standard Deviation

**Fig. 4:**
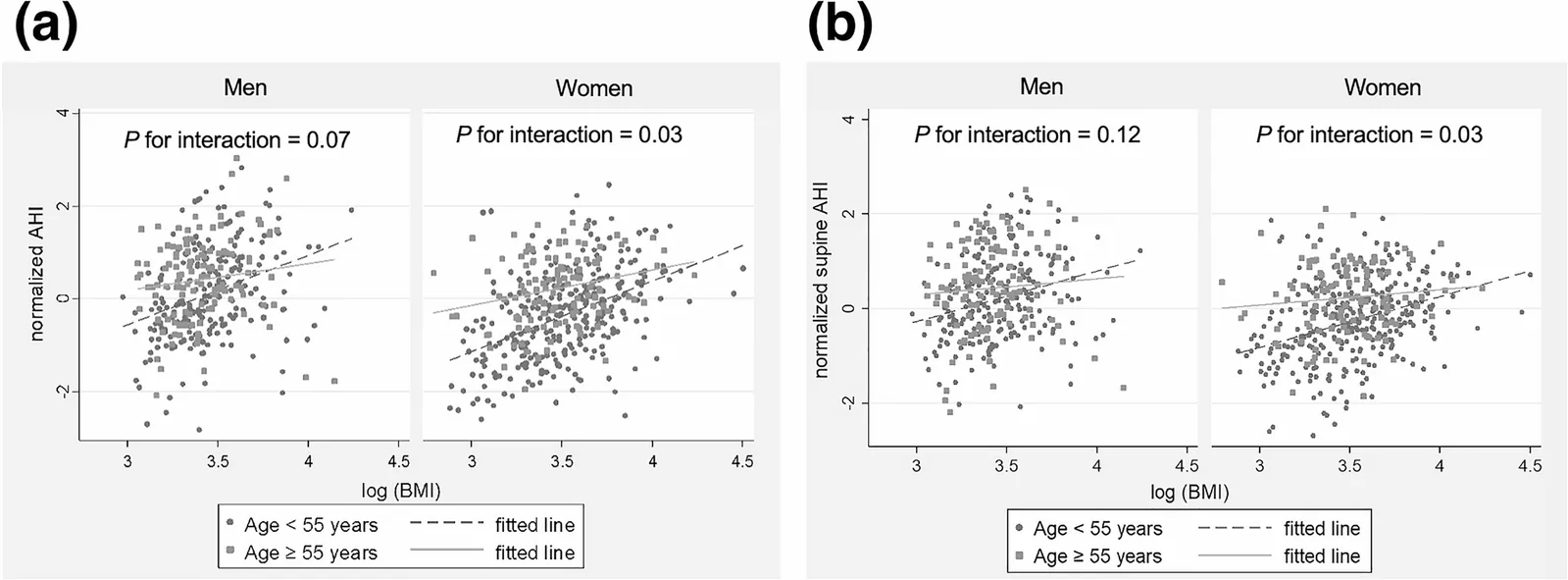
Normalized AHI and supine AHI vs. log(BMI) (**a**) The association between normalized AHI and log(BMI) according to sex and age. (**b**) The association between normalized supine AHI and log(BMI) according to sex and age. In women, a significant interaction between age group and log-transformed BMI was observed, indicating that the positive association between log(BMI) and AHI indices was weaker in older women compared with younger women. In men, no significant interaction was found

Additionally, there was a significant interaction between age group and log-transformed BMI in the association with normalized AHI and supine AHI in women (β for interaction = − 0.76, 95% CI − 1.46 to − 0.06, *P* = 0.03; and β = −0.75, 95% CI − 1.41 to − 0.09, *P* = 0.03, respectively), indicating that the positive relationship between log (BMI) and these indices was attenuated in older women compared with younger women. In contrast, no significant interaction was observed in men (β = −0.94, 95% CI − 1.94 to 0.07, *P* = 0.07; and β = −0.73, 95% CI − 1.67 to 0.20, *P* = 0.12) (Fig. 4). The log(BMI) × age group × sex three-way interaction was not statistically significant (normalized AHI: β = 0.17, 95% CI − 1.02 to 1.37, *P* = 0.77; normalized supine AHI: β = −0.01, 95% CI − 1.13 to 1.11, *P* = 0.98). However, in a sensitivity analysis defining older age as 50 years or older, the interaction was significant in men as well (normalized AHI: β = −1.49, 95% CI − 2.40 to − 0.58, *P* = 0.001; normalized supine AHI: β = −1.07, 95% CI − 1.92 to − 0.22, *P* = 0.01), whereas the three-way interaction with sex remained non-significant (*P* = 0.26 and 0.95, respectively) (Supplementary Fig. 3).

## Discussion

This study investigated whether the relationship between BMI and OSA severity varies according to age and sex. Our findings demonstrate that the association between BMI and OSA severity was attenuated in women aged 55 years or older compared with younger women. In younger women, higher BMI was associated with a greater proportion of OSA, whereas OSA prevalence among older women did not differ significantly across BMI groups. Similarly, the associations between BMI categories or log-transformed BMI and normalized total AHI and normalized supine AHI were significantly weaker in older women than in younger women. In men, the age-related interaction was not statistically significant in the main analyses, although the estimates were in the same direction, and some sensitivity analyses using an age cutoff of 50 years demonstrated significant age–BMI interactions in men as well. Moreover, the three-way interaction among BMI, age group, and sex was not significant. Thus, our data do not establish that the age-related attenuation differs between men and women. These findings suggest that the BMI–OSA relationship may weaken with age, particularly in women. Although this raises the possibility that factors other than BMI are relevant to OSA severity in older women, the cross-sectional design of this study does not allow us to determine the relative contribution of such factors.

Previous studies have consistently identified obesity as one of the strongest risk factors for OSA [2, 3] and have demonstrated increasing OSA prevalence with advancing age [5, 24]. However, the relationship between BMI and OSA in older adults appears to be more complex. Janssen reported that the prevalence of obesity-related health risks may differ in older populations, suggesting that the influence of excess body weight on disease outcomes may diminish with age [12]. These observations raise the possibility that the contribution of BMI to OSA severity may also vary across age groups.

Our findings extend this concept by suggesting that attenuation of the BMI–OSA relationship may be particularly evident among older women. While BMI remained strongly associated with OSA severity in younger women, its association with both total and supine AHI was weaker in women aged 55 years or older. Although the mechanisms underlying this observation cannot be determined from the present study, several age-related and sex-specific physiological changes may contribute.

One possible explanation is age-related changes in body composition and fat distribution. Older women tend to accumulate greater visceral and abdominal adiposity while simultaneously experiencing changes in upper airway structure and muscle function. Because BMI does not distinguish between lean mass and regional fat deposition, it may be a less accurate marker of obesity-related OSA risk in older women than in younger populations. This interpretation is consistent with prior studies questioning the utility of BMI as a comprehensive measure of adiposity in women undergoing age-related changes in body composition [25]. Alternative measures, such as waist circumference, waist-to-hip ratio, or imaging-based assessments of fat distribution, may better capture obesity-related OSA risk in this population.

The relationship between BMI and OSA severity may also be nonlinear, particularly at higher levels of obesity. Although our cohort was characterized by substantial obesity overall, the number of participants with BMI ≥ 40 kg/m² was relatively small in the older groups (Supplementary Fig. 4). Analyses using log-transformed BMI, which allow for attenuation of the relationship at higher BMI values, yielded findings similar to those based on BMI categories. Nevertheless, our ability to characterize the BMI–AHI relationship at extreme levels of obesity was limited, and the present study cannot determine whether potential nonlinearity at these levels reflects an AHI ceiling effect, obesity hypoventilation, or other mechanisms.

Hormonal changes occurring during the menopausal transition may also contribute. Declining estrogen and progesterone levels have been associated with reduced upper airway muscle tone and altered ventilatory control [10, 11]. Although menopausal status was not directly assessed in this study, these hormonal changes may partially explain the observed age-related differences among women. Future studies incorporating direct measures of menopausal status and reproductive hormones are needed to clarify the relative contributions of aging and menopause to OSA pathophysiology.

Previous studies have demonstrated that older adults exhibit greater positional dependency of OSA severity [16], with supine sleep leading to greater airway collapsibility and higher AHI compared to non-supine positions. Consistent with these findings, we observed age-related attenuation of the association between BMI and both total and supine AHI in women. These results raise the possibility that factors other than obesity, such as positional dependency, may contribute to OSA severity in older women and that treatment approaches beyond weight reduction may warrant evaluation in selected patients.

This study has several limitations. First, menopausal status and reproductive hormone levels were not available and therefore could not be directly assessed. Although the age threshold of 55 years was selected because it approximates the age by which most women have completed the menopausal transition [17], age cannot fully capture individual variation in menopausal status. Second, racial and ethnic data were not available for analysis. Because upper airway anatomy, body fat distribution, and OSA risk may differ across racial and ethnic groups [26], the generalizability of our findings may be limited. Third, because AHI and supine AHI were analyzed after rank-based inverse normal transformation, the regression coefficients are expressed in standard deviation units of the normalized scores rather than in events per hour, which limits their direct clinical interpretation. Fourth, the cross-sectional design precludes causal inference and does not allow assessment of the relative contribution of factors other than BMI, such as upper airway anatomy, regional fat distribution, and hormonal status, which were not measured. In addition, the number of older men was relatively small (*n* = 116), and the number of participants with BMI ≥ 40 kg/m² in the older age groups was small (12 men and 22 women), which may have limited the statistical power to detect age-related interactions in men and differences between sexes and increased sampling variability in the highest BMI category. Fifth, waist circumference, alcohol use, smoking status, and medication use were not available or were incompletely recorded. Therefore, residual confounding by these factors cannot be excluded. Sixth, hypopneas were scored according to the AASM guidelines in place at the time of the study, using the recommended criterion requiring ≥ 4% oxygen desaturation. Although this criterion was applied consistently across all participants and remains clinically relevant to Medicare coverage criteria, use of the current AASM recommended criterion incorporating ≥ 3% desaturation or an arousal would likely result in higher AHI values and could affect the observed associations. Therefore, whether the observed age-related modification of the BMI–OSA relationship would be similar using contemporary AASM scoring criteria remains uncertain. Finally, this study was conducted at a single university-based sleep laboratory and included patients referred for polysomnography, who may differ from the general population in OSA prevalence, severity, and comorbidity burden. Therefore, our findings may not be generalizable to community-based populations or other clinical settings. Future studies should incorporate direct assessment of menopausal status, body composition, and racial and ethnic diversity to better characterize age- and sex-related differences in OSA pathophysiology.

## Conclusions

This study identified age-related differences in the association between BMI and OSA severity among women. The association of BMI with OSA severity, including supine AHI, was attenuated in women aged 55 years or older compared with younger women. In men, the corresponding interaction was not significant in the main analyses. However, the formal test for a sex difference in this effect modification was also not significant.

Our results suggest that BMI alone may be a less informative predictor of OSA severity in older women. Given the attenuated relationship between BMI and both total and supine AHI in this population, weight reduction alone may not fully address OSA severity in some older women. Approaches targeting positional OSA, including positional therapy, may warrant evaluation as adjunctive treatment strategies in older women. Future longitudinal studies incorporating direct assessment of menopausal status, body composition, and hormonal factors are needed to clarify the mechanisms underlying these observations and to inform more individualized approaches to OSA evaluation and management.

## Supplementary Information

Below is the link to the electronic supplementary material.

Supplementary Material 1

## Data Availability

The datasets generated and/or analyzed during the current study are not publicly available. In accordance with Institutional Review Board approval, the data are restricted to the approved research team and may not be shared outside the team, including in deidentified, anonymized, or aggregated form. Accordingly, the data are not deposited in any public or restricted-access repository and are used solely for the purposes described in the IRB-approved protocol.
