# Supplementary material for: Age-dependent association of body mass index and obstructive sleep apnea severity in women
Source: Sleep Breath. 2026 Oct 31;30(6):302. doi: 10.1007/s11325-026-03849-y (PMC13723501; doi:10.1007/s11325-026-03849-y)
Supplement: Supplementary file 1 [file 11325_2026_3849_MOESM1_ESM.docx]

**Table S1.** Stata codes for creating a new variable, normalized AHI (norm_ahi), by using Rank-Based Inverse Normal Transformations (INT)

. gen ahi_ adj = ahi + uniform()*0.001

. egen rank_ahi = rank(ahi_adj), track

. gen percentile_ahi = rank_ahi / (_N+1)

. gen norm_ahi = invnormal(percentile_ahi)

**(a)**

**(b)**

*P* = 0.006

*

*

*

**

**

**Fig S1.** Proportion of OSA according to sex, age, and BMI (sensitivity analysis with an age cutoff of 50 years)

* *P* < 0.05 compared with BMI < 25 kg/m^2^ in men aged < 50 years. ** *P* < 0.05 compared with BMI < 25 kg/m^2^ in women aged < 50 years.

**(a)**

**(b)**

**Fig S2.** Normalized AHI and supine AHI according to BMI group (sensitivity analysis)

**(a)**

**(b)**

**Fig S3.** Normalized AHI and supine AHI vs log(BMI) (sensitivity analysis)

**Fig S4.** Scatter plots of raw AHI and supine AHI versus BMI according to sex and age group. (a) AHI (events/h) and (b) supine AHI (events/h) are plotted against BMI (kg/m²) without transformation. Circles and dashed lines indicate participants aged < 55 years and their linear fitted lines. Squares and solid lines indicate participants aged ≥ 55 years and their linear fitted lines.
